# Comparative genomics of European avian pathogenic *E. Coli* (APEC)

**DOI:** 10.1186/s12864-016-3289-7

**Published:** 2016-11-22

**Authors:** Guido Cordoni, Martin J. Woodward, Huihai Wu, Mishaal Alanazi, Tim Wallis, Roberto M. La Ragione

**Affiliations:** 1 Department of Pathology and Infectious Diseases, School of Veterinary Medicine, Faculty of Health and Medical Sciences, University of Surrey, Guildford, GU2 7AL UK; 2 Department of Food and Nutritional Sciences, University of Reading, Reading, UK; 3 Bioinformatics Core Facility, Faculty of Health and Medical Sciences, University of Surrey, Guildford, UK; 4 Ridgeway Biologicals Ltd, Units 1-3 Old Station Business Park, Compton, Berkshire RG20 6NE UK

**Keywords:** Avian Pathogenic *E. coli*, Virulence factors analysis, Multiplex PCR, Comparative genomics

## Abstract

**Background:**

Avian pathogenic *Escherichia coli* (APEC) causes colibacillosis, which results in significant economic losses to the poultry industry worldwide. However, the diversity between isolates remains poorly understood. Here, a total of 272 APEC isolates collected from the United Kingdom (UK), Italy and Germany were characterised using multiplex polymerase chain reactions (PCRs) targeting 22 equally weighted factors covering virulence genes, R-type and phylogroup. Following these analysis, 95 of the selected strains were further analysed using Whole Genome Sequencing (WGS).

**Results:**

The most prevalent phylogroups were B2 (47%) and A1 (22%), although there were national differences with Germany presenting group B2 (35.3%), Italy presenting group A1 (53.3%) and UK presenting group B2 (56.1%) as the most prevalent. R-type R1 was the most frequent type (55%) among APEC, but multiple R-types were also frequent (26.8%). Following compilation of all the PCR data which covered a total of 15 virulence genes, it was possible to build a similarity tree using each PCR result unweighted to produce 9 distinct groups. The average number of virulence genes was 6–8 per isolate, but no positive association was found between phylogroup and number or type of virulence genes. A total of 95 isolates representing each of these 9 groupings were genome sequenced and analysed for *in silico* serotype, Multilocus Sequence Typing (MLST), and antimicrobial resistance (AMR). The UK isolates showed the greatest variability in terms of serotype and MLST compared with German and Italian isolates, whereas the lowest prevalence of AMR was found for German isolates. Similarity trees were compiled using sequencing data and notably single nucleotide polymorphism data generated ten distinct geno-groups. The frequency of geno-groups across Europe comprised 26.3% belonging to Group 8 representing serogroups O2, O4, O18 and MLST types ST95, ST140, ST141, ST428, ST1618 and others, 18.9% belonging to Group 1 (serogroups O78 and MLST types ST23, ST2230), 15.8% belonging to Group 10 (serogroups O8, O45, O91, O125ab and variable MLST types), 14.7% belonging to Group 7 (serogroups O4, O24, O35, O53, O161 and MLST type ST117) and 13.7% belonging to Group 9 (serogroups O1, O16, O181 and others and MLST types ST10, ST48 and others). The other groups (2, 3, 4, 5 and 6) each contained relatively few strains.

However, for some of the genogroups (e.g. groups 6 and 7) partial overlap with SNPs grouping and PCR grouping (matching PCR groups 8 (13 isolates on 22) and 1 (14 isolates on 16) were observable). However, it was not possible to obtain a clear correlation between genogroups and unweighted PCR groupings. This may be due to the genome plasticity of *E. coli* that enables strains to carry the same virulence factors even if the overall genotype is substantially different.

**Conclusions:**

The conclusion to be drawn from the lack of correlations is that firstly, APEC are very diverse and secondly, it is not possible to rely on any one or more basic molecular or phenotypic tests to define APEC with clarity, reaffirming the need for whole genome analysis approaches which we describe here.

This study highlights the presence of previously unreported serotypes and MLSTs for APEC in Europe. Moreover, it is a first step on a cautious reconsideration of the merits of classical identification criteria such as R typing, phylogrouping and serotyping.

**Electronic supplementary material:**

The online version of this article (doi:10.1186/s12864-016-3289-7) contains supplementary material, which is available to authorized users.

## Background

Avian colibacillosis is an economically important infectious disease of domestic poultry [1, 2] and the responsible aetiological agent is *Escherichia coli*, with the most commonly implicated serotypes being O1:K1, O2:K1, O5, O8, O35, O150 and O78:K80 [3, 4]. The most severe clinical manifestation of *E. coli* infections in poultry is colisepticaemia, which often begins as an upper respiratory infection following a primary mycoplasmal or viral infection, leading to infiltration of the blood and internal organs and development of pericarditis, perihepatitis, airsacculitis and salpingitis [5]. Despite the worldwide importance of avian colibacillosis, there is still incomplete information regarding the genetic make-up of APEC.

Serogrouping and serotyping is an established tool to type APEC. Poxton (1995) and Bennett-Guerrero et al. (2000) demonstrated that core Lipopolysaccharides (LPS) and Lipid A of which there are 11 types determines protective immunity whereas the long chain of LPS defines the specificity of the immune response in part. In the studies of Dissanayake et al. (2008) core types R1-R4 were shown to be most prevalent within APEC using a ‘R-grouping by PCR’ technique [6–8]. However, describing only one factor of coliforms, the long chain of the lipopolysaccharide cell wall structure, serotyping and serogrouping is considered by many in the APEC field as of declining utility as a primary tool to describe APEC.

Phylogrouping is a top level genetic tool differentiating *E. coli* into larger clusters with each cluster representing either commensal groups or various pathotype groups. Devised by Clermont et al. (2000), it has been used for APEC (Gordon et al., 2008: Jakobsen et al., 2010a,b) who showed that APEC belonged predominantly to group B2 amongst others, but not the human associated group B3. Interestingly, group B2 is particularly well adapted to persistence in the hind gut of mammals [9–11]. There is evidence in the literature of good correlations between phylogroups and MLST, but not with serogrouping [12, 13]. For example, recent studies employing MLST have demonstrated that O8, a common serogroup that is often associated with colibacillosis, is comprised of many diverse genetic backgrounds with the majority belonging to the ST23 complex [14].

Whilst the tools described above are useful in partial characterisation of APEC further studies to define the specific virulence determinants encoded by them are required. The literature on the determination of pathogenicity is extensive and has been eloquently described by Dho-Moulin and Fairbrother [1]. Experimental chicken and turkeys models have been developed, permitting reliable evaluation of the pathogenicity of *E. coli* leading to the identification of many adhesins, iron sequestering systems, capsule, temperature-sensitive haemagglutinin, resistance to the bactericidal effects of serum and cytotoxic effects as virulence factors of APEC. Additional approaches such as subtractive hybridisation and random mutagenesis strategies [15] have identified other putative virulence genes and recently small and large plasmids have been implicated also [16, 17]. An interesting hypothesis generated out of the Nolan laboratory suggests that, although there are many disease presentations possibly arising through the expression of many combinations of virulence determinants, perhaps there exists a minimal set of genes that define APEC [18]. Maturana et al. (2011) used PCR to detect APEC virulence genes including *yjaA, tspE4.C2, iucA, irp-2, fepC, crl, csgA, tsh, lpfAO141, lpfAO154, iha, sitA, fyuA, fimA, papA* of which *crl, csgA, lpfAO141, lpfAO154, fimA, papA* and concluded that not all APEC strains have all determinants and different combinations of determinants in a strain will contribute to pathogenic potential [19].

Whilst the evidence presented above indicates that much is known of APEC, there remains a lack of clarity over their definition. Thus, this study aimed to further our understanding of the presence/absence of virulence factors in APEC and to attempt find congruence between factors used to identify APEC. In addition to add further depth to these analyses we investigated the genetic diversity of APEC from across Europe using whole genome sequencing (WGS).

## Methods

### Overview

In order to genetically characterise APEC isolates from Europe a panel of 272 isolates from across Europe was assembled and fully characterised using existing PCR approaches, but in a newly devised multiplex format targeting R type [8, 20], phylogroup [9], and 14 virulence factors [18]. The PCR data was then used to generate a similarity tree and facilitate the selection of 95 isolates for further whole genome analysis using NGS. The resultant genome sequences were assembled and subjected to Single-Nucleotide Polymorphisms (SNPs) analysis using APEC O78 (NC_020163) that facilitated the generation of a whole genome comparison tree. An additional ring map, generated by matching the sequences of each sample, at a nucleotide level, to the reference sequence (BLAST) [21] was produced and the top 500 high variable genes were compared for the 95 strains in order to confirm the results obtained using SNPs analysis.

### Strain selection and preparation for NGS analysis

#### Bacterial culture and DNA extraction

Samples were collected following ethical guidelines of the University of Surrey. Veterinarians that collected the samples also completed an accompanying submission form providing clinical data and laboratory results.

A total of 272 *E. coli* isolates from clinical samples collected from confirmed colisepticaemia cases (by *post-mortem* examination) were gathered from the UK (173), Germany (69) and Italy (30). Pure cultures submitted to the University of Surrey were streaked onto a suitable medium (typically Nutrient Agar or LB agar) and cultured for 16 h at 37 °C, aerobically. Following incubation a single colony was transferred into a 15 ml sterile Bijoux tube containing NB or LB broth and cultured again for 16 h at 37 °C with gentle agitation (225 rpm). Following incubation a 1 ml aliquot of the culture was transferred into a sterile tube and used for the DNA extraction. DNA was extracted and purified using ArchivePure DNA Cell/Tissue and Tissue Kits (5′Prime) according to manufacturer instruction and then quantified and stored at −20 °C. All APEC stock cultures were stored in HIB + glycerol at −80 °C.

#### Characterisation of APEC isolates using multiplex PCRs

The typing of APEC is complex and requires the use of a number of separate tests to ensure accurate isolate identification and characterisation. Here, three multiplex PCR tests (Tables 1, 2 and 3) were developed in order to facilitate the molecular typing of APEC investigating the presence/absence of: LPS core R typing, phylogrouping and virulence gene presence [9, 18, 20, 22]. All primer sequences and the conditions for PCR are also described in Tables 1, 2 and 3.

**Table 1:** 8plex, 5plex and 9plex primers and respective cycling conditions. The gene name, primer sequence and amplicon size are reported. Lowercase letters in the gene sequences (8plex) represent the tails added in order to obtain the same annealing temperature

8 PLEX		
Gene name	Sequence 5′–3′	Amplicon size bp
R1F +	gcgaaaaGAGTAATGTCGGGGCATTCA	551
R1R +	aggccaTTCCTGGCAAGAGAGATAAG	
R2F +	gcgaGATCGACGCCGGAATTTTTT	1141
R2R +	gcgagaAGCTCCATCATCAAGTGAGA	
R3F +	agccaGGCCAAAACACTATCTCTCA	1785
R3R +	agcgccGTGCCTAGTTTATACTTGAA	
R4F +	gcgcgcaTGCCATACTTTATTCATCA	699
R4R +	gcgcTGGAATGATGTGGCGTTTAT	
K12F +	gcaagTTCGCCATTTCGTGCTACTT	916
k12R +	acgcgcTAATCATAATTGGAATGCTGC	
chuAF +	aaatttgGACGAACCAACGGTCAGGAT	279
chuAR +	atttagTGTGAAGTGTCAGGAGACGCTG	
yjaAF +	aaaaaaCCGCCAGTACCAGGGACA	211
yjaAR +	gcagaaaaATGGAGAATGCGTTCCTCAA	
TSPEF+	gcgaaaaaGAGTAATGTCGGGGCATTCA	152
TSPER+	aaggCGCGCCAACAAAGTATTACG	

Cycling conditions: 95 °C for 5 min (Initial denaturation), followed by 2 cycles 95 °C (denaturation) for 30s, 50 °C for 30s (annealing), and 72 °C for 60s followed by 33 cycles 95 °C for 30s (denaturation), 58 °C for 30 s (annealing), and 72 °C for 60s (polymerization). On completion of 35 cycles the, a final polymerization was performed at 72 °C for 420 s

**Table 2:** 8plex, 5plex and 9plex primers and respective cycling conditions. The gene name, primer sequence and amplicon size are reported. Lowercase letters in the gene sequences (8plex) represent the tails added in order to obtain the same annealing temperature

5 PLEX		
Gene Name	Sequence 5′–3′	Amplicon size bp
iroN F	AATCCGGCAAAGAGACGAACCGCCT	553
iroN R	GTTCGGGCAACCCCTGCTTTGACTTT	
ompT F	TCATCCCGGAAGCCTCCCTCACTACTAT	496
ompT R	TAGCGTTTGCTGCACTGGCTTCTGATAC	
hlyF F	GGCCACAGTCGTTTAGGGTGCTTACC	450
hlyF R	GGCGGTTTAGGCATTCCGATACTCAG	
Iss F	CAGCAACCCGAACCACTTGATG	323
Iss R	AGCATTGCCAGAGCGGCAGAA	
iutA F	GGCTGGACATCATGGGAACTGG	302
iutA R	CGTCGGGAACGGGTAGAATCG	

Cycling conditions: 95 °C for 5 min (Initial denaturation), followed by 35 cycles at 95 °C for 30s (denaturation), 55 °C for 30s (annealing), and 72 °C for 40s (polymerization). On completion of 35 cycles, a final polymerization was performed at 72 °C for 420 s

**Table 3:** 8plex, 5plex and 9plex primers and respective cycling conditions. The gene name, primer sequence and amplicon size are reported. Lowercase letters in the gene sequences (8plex) represent the tails added in order to obtain the same annealing temperature

9PLEX		
Gene name	Primer sequence 5′–3′	Amplicon size
astA F	TGCCATCAACACAGTATATCC	116
astA R	TCAGGTCGCGAGTGACGGC	
irp2 F	AAGGATTCGCTGTTACCGGAC	413
irp2 R	AACTCCTGATACAGGTGGC	
papC F	TGATATCACGCAGTCAGTAGC	501
papC R	CCGGCCATATTCACATAA	
iucD F	ACAAAAAGTTCTATCGCTTCC	714
iucD R	CCTGATCCAGATGATGCTC	
tsh F	ACTATTCTCTGCAGGAAGTC	824
tsh R	CTTCCGATGTTCTGAACGT	
vat F	TCCTGGGACATAATGGTCAG	981
vat R	GTGTCAGAACGGAATTGT	
cvi/cva F	TGGTAGAATGTGCCAGAGCAAG	1181
cvi/cva R	GAGCTGTTTGTAGCGAAGCC	
ibeA F	AGGCAGGTGTGCGCCGCGTAC	171
ibeA R	TGGTGCTCCGGCAAACCATGC	
sitA F	AGGGGGCACAACTGATTCTCG	608
sitA R	TACCGGGCCGTTTTCTGTGC	

Cycling conditions: 95 °C for 5mins (initial denaturation), followed by 35 cycles at 95 °C for 30s (denaturation), 55 °C for 30 s (annealing), and 72 °C for 120 s (polymerization) On completion of 35 cycles, a final polymerization was performed 72 °C for 600 s

The first multiplex is an 8-plex and the primers target LPS core, R typing and phylogrouping genes. A tail was added to the 5′ end of primers used in prior literature [9, 20] to obtain a common annealing temperature facilitating the multiplexing. The other two multiplexes (5 plex and 9 plex) targeted a panel of common APEC virulence genes [18]. Gel electrophoresis was performed using a 2% gel for the 8 and the 9 plex and a 3% gel for the 5 plex (to enhance the amplicon separation) at 130 V and 65A for 40 min. The presence/absence of all the genes investigated using the multiplex PCRs were marked as G = positive and A = negative in order to facilitate the use of MEGA software [23] (using the default options and UPGMA linkage). DNA from selected samples was sequenced using Illumina platform (Mi-seq) at the Animal Health Trust (AHT) laboratories, Newmarket, UK.

#### Investigating virulence factor associations by using data mining and machine learning approaches

A preliminary study using machine learning and data mining software WEKA was conducted in order to understand if the virulence factors found in APEC could be inter-dependant (e.g. IF value 1 is true THEN also value 2 is true) [24]. Using this tool we looked at virulence factor association using the Apriori algorithm leaving all the parameters as per the default [25].

### Next generation sequencing

Extracted DNA was quantified using Qubit® dsDNA BR Assay Kit (ThermoFisher Scientifics) following the manufacturer instructions. The concentration of DNA was adjusted to 20 ng/μl in sterile MilliQ water and sent to the Animal Health Trust (AHT) for sequencing. At the AHT libraries were constructed using Nextera DNA Library Preparation Kit (Illumina) following the manufacturer’s instructions. NGS analysis was performed using a MiSeq next-generation sequencer using paired ends method. For each isolate approximately 1 million 150 bp short reads were obtained. Sequence lengths, ranging between 4.6 M and 5 M which represents almost the entire genome length of *E. coli* were obtained.

#### *De novo* assembly

The raw sequences obtained were *de novo* assembled in contigs using the software Velvet (Additional file 1) [26–28] and the Vague graphical user interface (GUI) [29]. The K-mer size was individually chosen by instructing the software to calculate the size according to a genome of 4.7 M bases (according to the number of base pair of the reference strain APEC O78 NC_020163). The *de novo* assembled contigs were then ordered against NC_020163 using the software Mauve [30] using the contigs alignment option and allowing multiple cycles of aligning (until the computer provided the final alignment).

#### Annotation and analysis of the results

The ordered contigs were submitted to the RAST server for the automatic annotation of the genome features following the suggested guidelines (default options) [31]. In addition to the Genbank file (full annotated scaffold) RAST generates a new multi-FASTA file containing the original contigs submitted and split according to the features found.

These files were downloaded and using the online tools provided by Center for Genomic Epidemiology (http://www.genomicepidemiology.org/), used for the following analysis:

- Single-nucleotide substitutions (polymorphisms) (SNPs) analysis (reference sequence APEC O78 (NC_020163))
- Antimicrobial resistance (AMR) (default configurations)
- Multi-locus sequence typing (MLST)
- Serotype (default configurations)

**Fig. 1:**
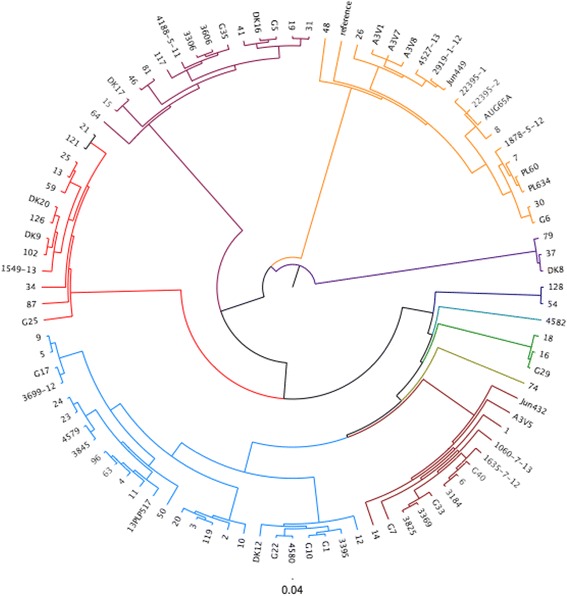
Cladogram based on the 22 virulence factors analysed using multiplex PCRs. Based on this analysis nine main groups were identified and are highlighted in different colours. The branches of the tree are proportional to the distance between the strains. Legend: group 1 = dark green, group 2 = yellow, group3 = blue, group 4 = light green, group 5 = red, group 6 = dark yellow, group 7 = turquoise group 8 = dark blue, group 9 = dark red

In order to explore the genetic diversity between the 95 strains analysed, a whole genome Single-Nucleotide Polymorphisms (SNPs) analysis was performed using the online software CSI Phylogeny 1.0a [32]. Here, the analysis focuses upon all the genes shared in common by the strains and the number and location of the single nucleotide differences within those common genes. This provided a detailed overview of deeper phylogenetic relatedness. To facilitate this the strains were investigated for SNPs and compared to the reference APEC O78 strain deposited in the NCBI database (NC_020163). This analysis facilitated the grouping of strains according to their overall similarity with the O78 reference strain, as reported in Fig. 1, where the branches of the 10 groups found are highlighted in different colours.

**Fig. 2:**
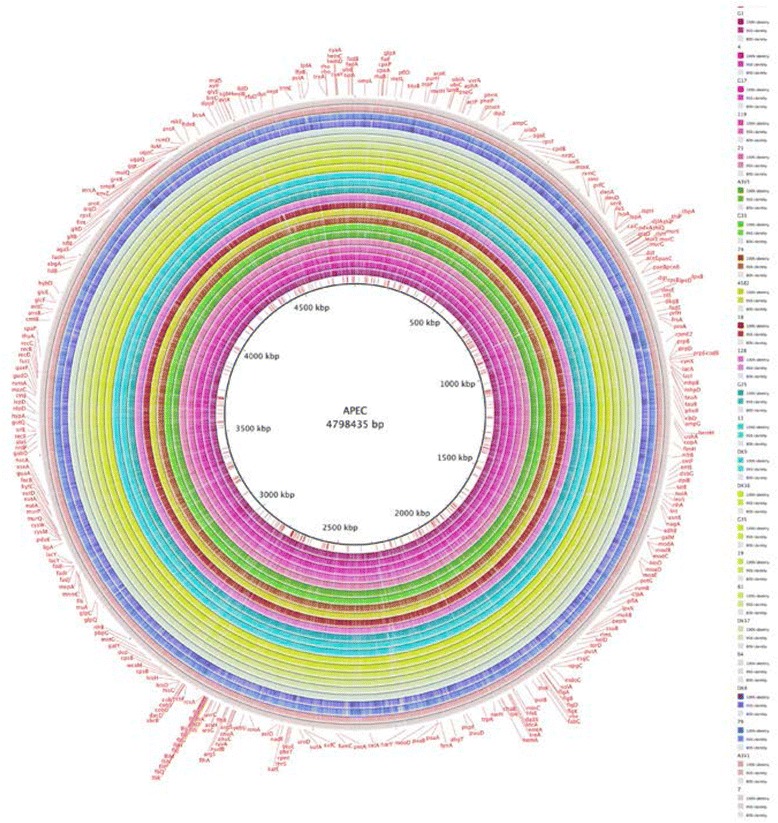
Ring map. Using the groups determined using the SNPs analysis, sequences from each group. The sub-groups were compared with the APEC O78 Genebank file. The legend on the side illustrates the colour adopted for each strain. Strains belonging to the same group have the same colour (different tones in order to distinguish each strain). The annotation reported on the external ring is the one downloaded from NCBI database (APEC O78 Genebank file)

In order to verify the SNP tree, FASTA sequences of samples from each group (and sub-groups) were compared with the database APEC O78 using the software Blast Ring Image Generator (BRIG) [33]. Each group was colour coded and the sub-groups were represented as a hue of the same group colour (e.g. purple, fuchsia, pink or red, orange yellow) (Fig. 2).

As the strains analysed were presumed to be closely related (all APEC strains), the threshold for the colour coding was adjusted as follow: the full colour area representing the part of the genome having between 95 to 100% similarity with O78 strain NC_020163. The half coloured areas indicating similarities that range between 80 to 95% and the white area represent the areas of the genome with similarities below 80%.

The SNPs analysis data (Nenwick file) were also loaded into the software FIGtree for presentation purposes (groups colour coding) [34].

**Fig. 3:**
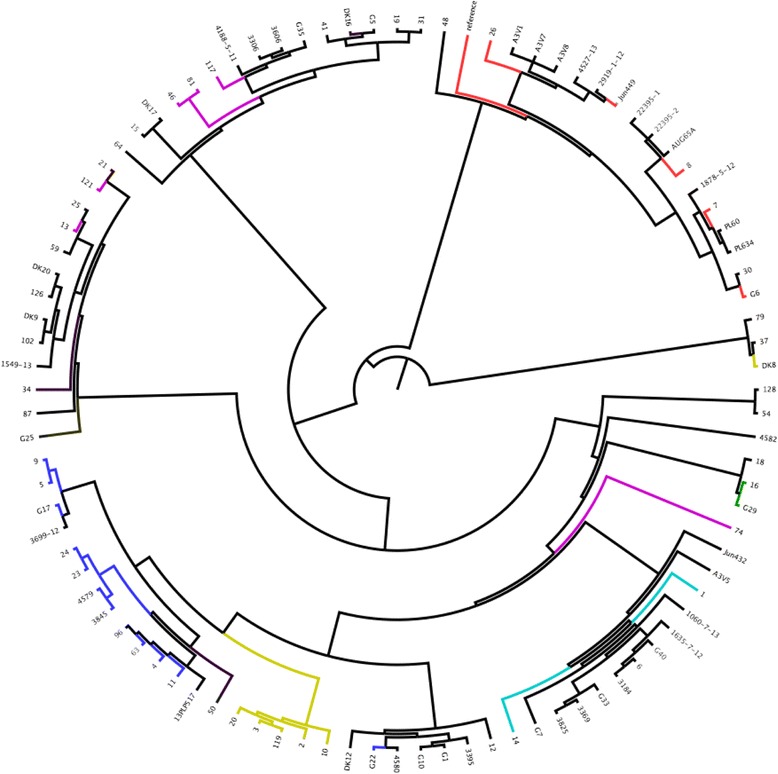
SNPs analysis of 95 strains analysed. O78 was used as reference strain. Each group found was highlighted with a different colour. Legend (clockwise order): Group1 = yellow, Group2 = mauve, Group3 = purple, Group4 = turquoise, Group5 = green, Group6 = mustard yellow, Group7 = brown, Group8 = blue, Group9 = red, Group10 = Magenta

**Fig. 4:**
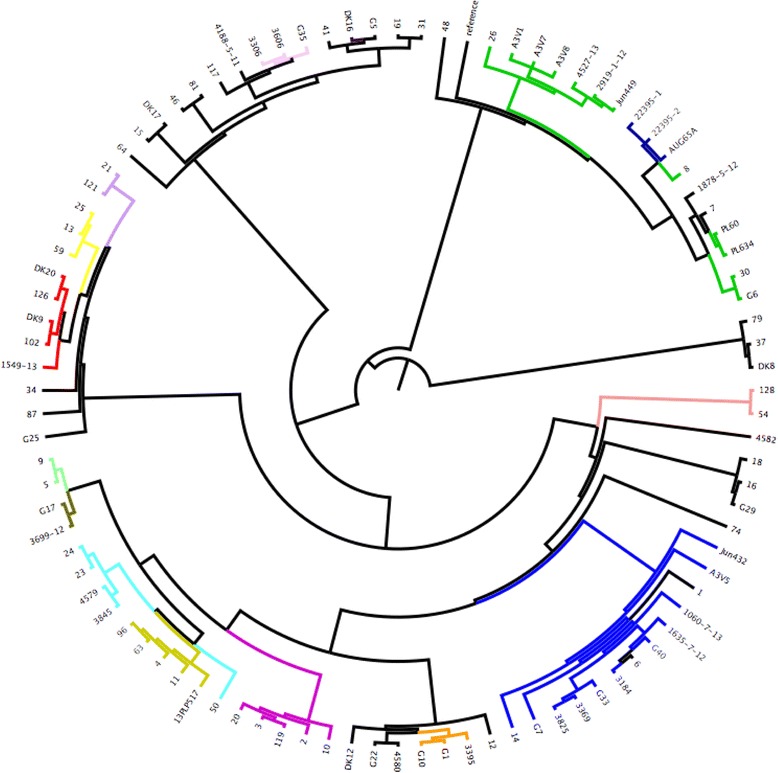
Serotypes correlation with SNPs tree. Only O type found in more than one isolate. Legend: Red = O78, Purple = O8, Blue = O2, Turquoise = O24, Gree*n* = O15, yellow = O4. Black = not determined

**Fig. 5:**
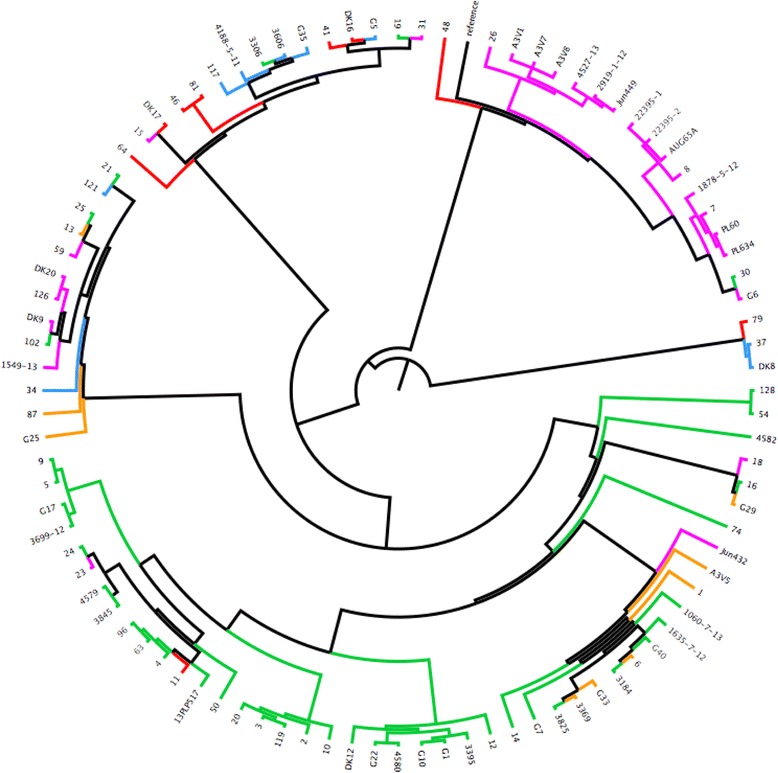
MLST data colour coded and matched with the SNPs tree. Legend: Red = ST10, Gree*n* = ST23, yellow = ST48, Salmon Pink = ST57, Cya*n* = ST95, Pink = ST101, Blue = ST117, Amber = ST140, Light gree*n* = ST141, Brow*n* = ST355, Orange = ST428, Light mauve = ST746, Purple = ST1618, Dark blue = ST2230

Data from phylogrouping, MLST and serotype were also used to colour-code the SNPs generated tree (Figs. 3, 4 and 5).

From each SNPs and sub-group identified, one strain was selected and loaded into BRIG software in order to generate a ring map (BLAST based) [21, 33] (Fig. 2). This analysis aimed to further confirm the validity of the SNPs grouping and to identify areas of the genome where divergence was evident.

All the data from multiplex PCRs, SNPs groups, MLST, serotype, AMR, R-type, phylogrouping were merged into an Excel sheet (Additional file 2) for further statistical analysis (using Excel or SPSS software) with the purpose of identifying correlations between these data sets, with a particular emphasis on identifying any geographically meaningful distributions.

#### Genome similarity analysis of strains

Blast matching data obtained using the software BRIG were used to perform the best reciprocal BLAST hits analysis choosing a conservative value of E- < e-7 [35, 36]. That means that for a gene, hit percentage is defined as number of nucleotides covered by the strain reads divided by the total number of nucleotides of this gene. Only that strain reads having E-value < e-7 in terms of blast outputs are used for hit percentage calculation. So hit percentage = 1 means that the strain perfectly contain this gene; and hit percentage = 0 means that the strain doesn't contain this gene.

**Fig. 6:**
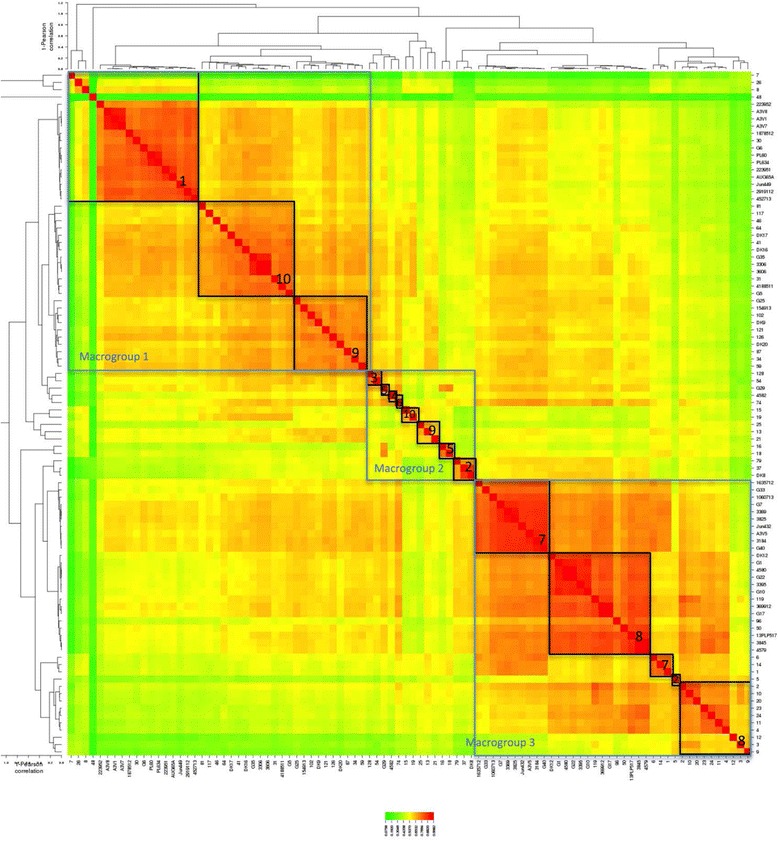
Phylogrouping data correlation with SNPs tree. Legend: Turquoise = A, Purple A1, Red B1, Green B2, Orange D. Groups A1 and B2 tend to cluster correctly, while the others (A, B1 and D) can be found mixed accross different groups

Using these data (available in the file his.xlsx in supporting material) heat maps were generated (Additional file 3 and Fig. 6) in order to explore the convergence of results the groups found using the SNPs analysis. In order to obtain the heat map in Fig. 6 a similarity matrix was obtained and the data were plotted using the online tool CIMminer (http://discover.nci.nih.gov/cimminer. accessed on 20/10/2015) using Pearson Correlation method.

## Results and discussion

### Analysis of 272 strains using multiplex PCRs

#### LPS core R typing by PCR

LPS core R typing indicates that the vast majority of APEC strains belong to R1 type (55%), followed by R3 (32.85%), R4 (27.1%), and R2 (6.5%). Tables 4 and 5 reports the strains that were positive for more than one R type or negative for all R types. In line with previous findings [20, 37–40], here we showed that R1 was the most common type, but interestingly of the 272 APEC strains analysed, 74 were positive for multiple R types and 5 were negative for all R types investigated in this study (Tables 4 and 5). This does not imply a lack of LPS core genes, but possession of as yet undefined types. Fortunately, these were a very small minority of the strains examined and thus do not confound our findings, but further studies should be undertaken to assess the LPS core of these undefined strains as a new LPS core (if any) will impact on *E. coli* phylogeny.

**Table 4:** Multiple R type and un-typable strains list

Sample N	R1	R2	R3	R4	K12
2	+	-	+	-	-
3	+	-	+	-	-
5	+	-	+	-	-
8	+	-	+	-	-
9	+	-	+	-	-
10	+	-	+	-	-
12	+	-	+	-	-
19	+	-	+	-	-
21	+	-	+	-	-
22	+	-	+	-	-
24	+	-	+	-	-
25	+	-	+	-	-
26	+	-	+	-	-
29	+	-	+	-	-
30	+	-	+	+	-
31	+	-	+	-	-
32	+	-	+	-	-
33	+	-	+	-	-
35	+	-	+	-	-
44	+	-	+	-	-
63	+	-	+	-	-
65	+	-	+	-	-
66	+	-	+	-	-
68	+	-	+	-	-
74	+	-	+	+	-
76	+	-	+	-	-
77	+	-	+	-	-
78	+	-	+	+	-
79	-	-	-	-	-
81	+	-	-	+	-
83	+	-	+	-	-
85	+	-	+	-	-
86	+	-	+	-	-
90	+	-	+	-	-
96	+	-	+	-	-
97	+	-	+	-	-
98	+	-	+	-	-
99	+	-	+	-	-
100	+	-	+	-	-
101	+	-	+	-	-
103	+	-	+	-	+
104	+	-	+	-	-
105	+	-	+	-	-
106	+	-	+	-	-
112	+	-	+	-	-
114	+	-	+	-	-
116	+	-	+	-	-
119	+	-	+	-	-
120	+	-	+	-	-
122	+	-	+	-	-
125	+	-	+	-	-
129	+	-	+	-	-
A3V-3	+	-	+	-	-
A3V-4	+	-	+	-	-
A3V-9	+	-	+	-	-
3699-12	+	-	+	-	-
3603	-	-	+	+	-
3340	+	-	+	-	-
3397	+	-	+	-	-
3398	+	-	+	-	-
3606	-	-	+	+	-
3848	-	-	+	+	-
4582	-	-	-	-	-
4578	+	-	+	-	-
4579	+	-	+	-	-
G8	-	-	+	+	-
G34	-	-	+	+	-
G35	-	-	+	+	-
G39	-	-	+	+	-
G40	-	-	-	-	-
G43	+	-	+	+	-
G44	-	-	-	-	-
G46	-	-	+	+	-
G48	-	-	+	+	-
DK2	-	-	+	+	-
DK5	-	-	-	-	-
DK6	-	-	+	+	-
DK8	-	+	-	-	+
DK16	+	-	+	-	-

**Table 5:** Phylogrouping (frequency) divided by Country

Country	Total N.	A	A1	B1	B2	D
DK	68	11 (16..2%)	13 (19,1%)	10 (14,7%)	24 (35,3%)	10 (14,7%)
IT	30	3 (10,0%)	16 (53,3%)	0 (0,0%)	10 (33,3%)	1 (3,3%)
UK	173	23 (13,3)	30 (17,3%)	12 (6,9%)	97 (56,1%)	11 (6,4%)

An attempt was made to correlate the presence of virulence factors with the R type, but none was established. Similarly, it was not possible to correlate virulence factor carriage, O type or MLST data. Therefore, it was not possible to associate LPS core type in the pathogenesis of APEC and this is perhaps not surprising as acquisition and loss of virulence factors is relatively dynamic compared with genes encoding core functions that are likely to have selective pressure upon them to remain invariant. R type is likely to reflect deeper phylogeny than ephemeral factors such as virulence and antibiotic resistance genes for example. However, the data strongly indicate that R typing alone cannot be used to discriminate between different APEC isolates (Additional file 2) as might be predicted from prior studies [20–23].

#### Phylogrouping and enumeration of virulence genes by PCR

**Fig. 7:**
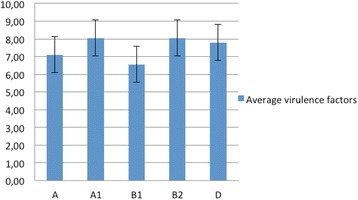
Comparison with the groups obtained using SNPs analysis (coloured names) and the groups obtained using multiplex PCRs. Legend: group 1 = red, group 2 = mocha, group 3 = salmon pink, group 4 (1 sample) = bright green, group 5 = lavender, group 6 (1 sample) = sky blue, group 7 = dark green, group 8 = blue, group 9 = dark red, group 10 = mauve

**Fig. 8:**
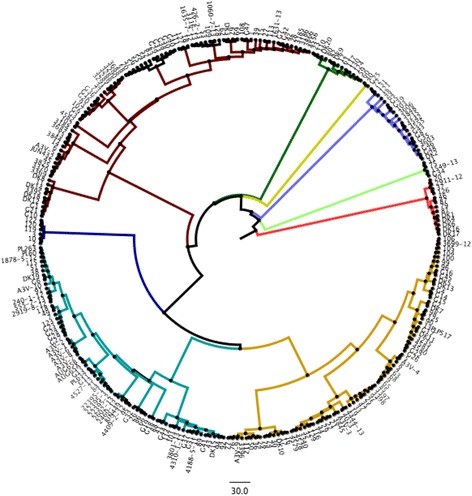
The mean and the standard deviation of the total number of virulence factors detected in each phylogroup (A, A1, B1, B2, and D)

The phylologroups A, B1, B2, D were determined in 2000 by Clermont [9] using the dichotomous approach that was enhanced by the addition of new subgroups described in 2010 by Carlos et al. [41]. Those were the groups A1, B3 (only found in humans) and D2 [41]. Using this existing classification [9, 41] for the 272 strains analysed we found (in decreasing order) that 132 grouped in B2 phylogroup, 61 in A1, 37 in group A and 21 in groups B1 and D. The remaining 21 were not ascribable to any group. Interestingly, previous studies by Walk et al. [42], demonstrated that the majority of *E. coli* strains that are able to persist in the environment belong to the B1 phylogenetic group. As relatively few of the strains examined here belonged to this ‘environmental’ group we can probably conclude these strains were less likely to be opportunistic pathogenic *E. coli* associated with, but not necessarily causing avian colibacillosis. No B3 (human only) strains were found, confirming host differentiation, a finding consistent with the incorrect view that APEC were associated with urinary tract infections in man that arose through dependence on analysis of carriage of some shared virulence determinants by UTI strains [4, 18, 22, 43, 44]. Johnson et al. [45] found that strains from phylogroups B2 and D harboured more virulence factors than strains from the phylogroups A and B1 [41], but the studies reported here differ as the average value of the virulence factors ranging between 6 to 8 factors was common to all the phylogroups found (Fig. 7). The average and the standard deviation of the number of virulence factors detected for each phylogroup is illustrated in Fig. 8. In the studies conducted in our laboratories we have noted that the carriage of virulence determinants (up to 5 maximum), by presumed commensal strains (not belonging to recognized APEC serotypes,unpublished findings) is notable in European avian *E. coli* isolates.. The Nolan laboratory [18] previously suggested that the detection of a minimum of 5 virulence factors could be used to define APEC, but the data produced here suggests this number is perhaps too low. Therefore, here we shall discuss other factors that must be considered before a definition of APEC can be authoritatively assigned to an isolate.

Interestingly, Extra Intestinal Pathogenic *E. coli* (ExPEC) strains frequently belong to phylogroups B2 and D, the commensal strains to groups A and B1, whilst the intestinal pathogenic strains belong to groups A, B1 and D [41].

When analysed by country, the German, Italian, and British strains, showed notable differences in the distribution of phylogroups. In fact 35.3% (*p* = 0.002) of German strains belonged to phylogroup B2, while in Italy 53.3% (*p* = 5.24e-5) belonged to phylogroup A1 and in the UK 56.1% (*p* = 1.72e–25) of the strains belonged to phylogroup B2. These differences were significant (*p* < 0.05). (Table 5 and Additional file 2.

#### Understanding APEC diversity: 22 factor based cladogram

Data from R typing, phylogrouping and PCR data covering 14 virulence genes was used to generate a cladogram based on the presence/absence of the factors investigated. On the basis of the 22 factors investigated with each factor given equal weighting the strains grouped in 9 major groups, as showed in Fig. 2. The analysis of the 22 factors was used also as a tool to select the strains for further analysis using NGS approaches (see below) and to attempt classification of APEC isolates but no correlations between R type, phylogroup and number of virulence factors analysed could be deduced (full dataset is available in the Additional file 2). The conclusion to be drawn from the lack of correlations is that firstly APEC are very diverse and secondly it is not possible to rely on any one or more of the tests to define APEC with clarity reaffirming the need for whole genome analysis approaches which we describe here.

#### Investigating virulence factor associations by using data mining and machine learning approaches

We found strong associations (confidence 0.99) between:

1. ompT (protease) == > hylP (haemolysin)
2. IroN (siderophore) + ompT (proteases) == > hlyp (haemolysin)
3. ompT (proteases) + sitA (cell adhesion–metal ions binding== > hlyp (haemolysisn).
  Lower, but yet statistically significant factor association were also found for
4. IroN (siderophore) + sitA (cell adhesion–metal ions binding) == > hlyP (haemolysisn conf:(0.96)
5. cva/cvi (bacteriocin immunity) == > hlyP (haemolysisn) conf:(0.96)
6. hlyP (haemolysisn) + sitA (cell adhesion metal ions binding) == > iron (siderophore) conf:(0.95)
7. hlyp(haemolysisn) = 1 sitA (cell adhesion–metal ions binding) == > ompT (proteases) conf:(0.95).

### Comparative genomics using NGS (selected isolates only)

**Fig. 9:**
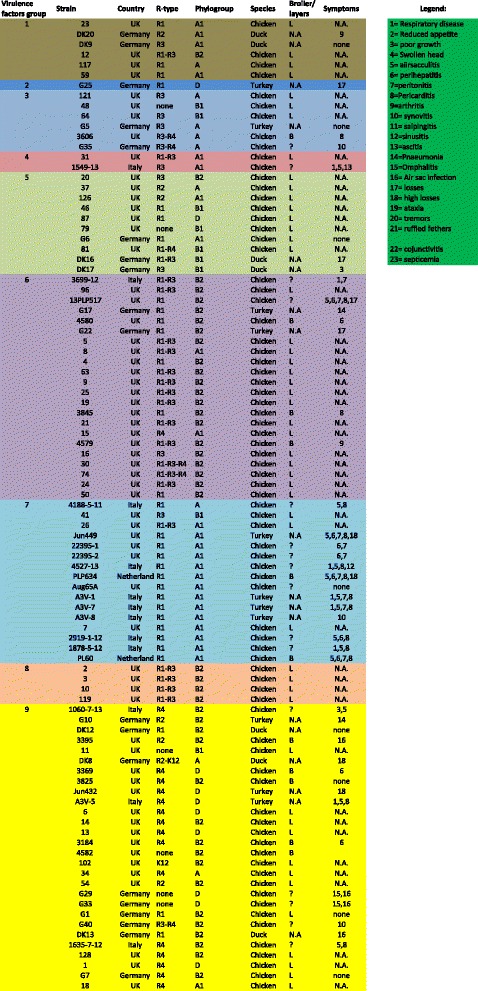
Heat map of the similarity matrix obtained from best reciprocal BLAST hits values (Pearson correlation). Colour shades indicate the percentage of correlation (1 = 100%): from red to yellow 0.9982 to 0.5370; from yellow to green 0.5370 to 0.0758. The groups found with SNPs analysis have been highlighted (black squares). Analysis of the colours illustrates three distinct macro-groups (blue square). Macro-group 1 containing isolates SNPs groups 1,10 and 9; macro-group 2 containing isolates SNPs group 2,3,4.5.6.9,10 and macro-group 3 containing isolates from SNPs groups 2,7, and 8. Noteworthy is the fact that macro-group 2 contains strains from both the other macro-groups, suggesting that the strains in this macro-group could be either ancestors or newly evolved strains

From each of the 9 groups and sub-groups determined using the 22 factor multiplex PCRs, 95 APEC strains were selected to cover the groups and diversity within each of the groups and submitted for whole genome sequencing: the subgroups, R type, phylogroup, country of origin, species, broiler/layer and clinical symptoms reported were also considered when selecting the panel of strains for NGS (Fig. 9 summarises the strain selected).

A preliminary visual analysis of the ring maps obtained, showed that within the groups, the patterns of similarities/differences (coloured/white areas) are conserved and only minor variation from the pattern were visible and referable to the sub-groups of origin of the samples included.

The convergent similarities within groups and the differences between the groups evidenced with this analysis confirm the accuracy of the tree generated using SNPs using two different methods (BLAST analysis of single nucleotides and SNPs) comparable results were obtained. In order to quantify this early visual analysis, the best reciprocal BLAST hits analysis (E < e − 7) was performed.

The whole genome SNP analysis of shared genes gives a deep phylogenetic picture and it is now possible to undertake pairwise analyses of whole genome SNP analysis with all other types of strain definition and following sections of this paper deal with this in depth.

#### Comparison between R-type and SNPs grouping

R-type correlation was attempted in order to verify if R-typing could be a suitable method to characterise APEC. However, according to our data (Fig. 9) this is possible in some cases (e.g. group 7 and 8) where there is consensus between the R-type (R1 and R1-R3), but this does not exclude the presence of the same R-types in the other groups. Hence we can conclude that the R-typing alone is not a suitable method for APEC characterisation [8].

#### Comparison between phylogroup and SNPs grouping

An attempt to correlate the data from phylogrouping with the whole genome SNPs tree was made. Many strains joined to the same groups according to their phylogroup, but for each phylogroup considered there were also strains that belonged to the same phylogroups, but clustered in different branches of the SNPs tree (as shown in Fig. 6 (e.g. sample 10, 11, 16, 18, G29 and 23)). These data indicate that phylogrouping alone may not be the ideal test for discriminating between APEC strains. This was also previously reported by Gordon et al. 2008 who found that only 80–85% of the phylogroup memberships assigned using the Clermont method were correct [46]

#### *In silico* Serotyping and MLST comparison with SNPs grouping

Using the online software tool, SeroTypeFinder, MLST 1.0 [47] Resfinder [48], it was possible to determine the serotype, MLST group and antibiotic resistances of sequenced strains (see Additional file 2), but due to the uneven distribution of the samples analysed, it was not possible to prove statistically that the different correlated with country of origin. So the apparent distribution of the serogroups and MLST across Europe, indicating that a greater variability in both serotype (*n* = 23) and MLST (*n* = 25) exists in the UK between the APEC strains compared to Germany (*n* = 8; *n* = 14) or Italy (*n* = 1; *n* = 6) cannot be statistically confirmed.

A total of 23 different serogroups were found and 46 isolates were un-typable; and 34 different ST types were found and (10 strains were un-typable). The serotype and MLST data are reported in Additional file 2.

Serotyping and MLST data obtained were compared with the tree generated from the SNPs to verify if the deduced serotypes/MSLT were correlated to the groups found using the SNPs analysis. In Fig. 4, O types (the ones reported in more than one sample) were highlighted in the tree. The analysis of this data indicated that each O type was associated with a group found using the SNPs analysis with the notable exceptions of the O8 (purple) group that was distributed across three different groups (6, 9 and 10) and the DK8 strain (O4 serotype) which was associated with a different SNPs group (2 instead of 8). Apart from these two exceptions all the other serotypes belonged to a single group and in detail: O78 joined group 1, O15 group 5, O24 group 7. Both O2 and O4 merged in group 8.

Data obtained from MLST analysis was also matched with the tree obtained using the whole genome SNPs. This is an analysis based on a subset of 8 highly conserved genes and it might be anticipated to be highly correlated with whole SNP analysis. This was found to be the case. The data confirmed that the MLST groups correlated with the O typing tree. Considering the different ST types and their association with the whole genome SNPs groups (Fig. 5) it is possible to see that group 1 was populated mainly by ST23 and three samples belonging to ST2230; group 3 ST57; group 7 was joined by the sole ST117; group 8 resulted to be a mix of ST428, ST1618, ST95, ST140 and ST141; group 8 was formed by ST10, ST48 and ST746; and finally the group 10 was joined by ST101. Groups 2, 4, 5 and 6 were not joined by any ST detected more than one time in our analysis so these data were discarded.

The analysis of the distribution of the ST-types in the different countries included in the study indicated that whilst ST10, ST23, and ST117 were isolated in all the countries included in the study, whereas the other ST-types were only found only in some (Table 6).

**Table 6:** Presence of ST types in Germany (DK), Italy (It) and United Kingdom (UK). In each column the ST types that were found in only one country are reported. The results included ST101 and ST428 (present in DK and UK, but not IT), and ST355 (present in DK and IT, but not in the UK)

Germany	Italy	UK
69, 93, 101 (also in UK), 131, 133, 355 (also in Italy), 428 (also in UK), 661, 1326, 1582, 1611.	355, 269, 602	46, 57, 95, 101, 140, 141, 155, 297, 388, 428, 429, 696, 746, 770, 1056, 1114, 1276, 1304, 1618, 1638, 2230, 3578.

#### *In silico* Antimicrobial Resistances (AMR) analysis

One of the major health issue associated with *E. coli* is its role in the emergence and the dissemination of antimicrobial resistance [49]. Most of the resistance properties emerge from commensal bacteria in the gastrointestinal tract [50] where bacteria exist at a high density, allowing horizontal resistance gene transfer between strains from a single species and/or between species or even genera. Therapeutic practices in humans and domestic animals that involve use of antimicrobial agents, allow for the selection of resistant strains [51]. One of the mechanisms involved in the spread of antimicrobial resistance is the emergence of some specific clones that acquire resistance genes, mostly via mobile genetic elements such as gene cassettes, transposons, integrative genetic elements, and plasmids and that due to an increase in fitness become widespread [52, 53].

Identification and analysis of AMR genes was conducted using the online software Resfinder [48]. This online tool uses a database of more than 2,000 resistance genes covering 12 types of antimicrobial resistance agents (aminoglycoside, beta lactamase, fluoroquinolone, fosfomycin, fusidic acid, glycopeptide, macrolide-lincosamide-streptograminB, phenicol, rifampicin, sulphonamide, tetracycline, and trimethoprim) [54]. The results of this analysis indicated that the 51.7% (49/95) did not carry any of the 2000 genes investigated. However, the other 48.3% (46/95) of the strains showed single or multiple genes encoding resistance: Tetracycline (34%), Beta lactamase (30%), Aminoglycosides (21%), Sulphonamide (20%), Phenicol (7.4%), Fluoroquinone and MLS (Macrolide-Lincosamide-Streptogramin B) (1%). No strain showed resistance to Fosfomycin, Fusidic acid, Nitroimidazole, Oxazolidinone, Rifampicin, Trimethoprim or Glycopeptide. The genes involved in antibiotic resistance are reported in Table 7. These data will need to be confirmed phenotypically by MIC testing of appropriate isolates as in extraintestinal *E. coli*, multi drug resistance (MDR) is most commonly associated with plasmids and, moreover, this analysis only refers to genomic DNA [55]. The analysis to date has not considered mutational AMR such as resistance to the flouroquinolones as these are encoded usually by chromosomal genes that have mutated [56]. With regard to quinolone resistance it is possible to look at the *gyrA* gene and assess changes in the quinolone resistance-determining regions (QRDR) where resistance mutations arise [57].

**Table 7:** List of the resistance genes found following analyses of the 95 sequenced APEC strains. Similar genes names (e.g. aadA1 and aadA2) have been reported, including the first part of the name (e.g. aadA1, A2)

Antibiotic	Gene
Aminogl.	aadA1,A2; strA,B; aph(3′)-Ia,
Beta-Lac.	blaTEM-1A, 1B, 1C,1D, CTX-M-1, CMY-2
Fluoroquin.	qnrB19
Fosfomycin	none
Fusidic Acid	none
MLS	mph(B)
Nitroimidazole	none
Oxazolidinone	none
Phenicol	cat(A1)
Rifampicin	none
Sulphonamide	sul1, 2
Tetracycline	tet(A), (B)
Trimethoprim	none
Glycopeptide	none

#### Matching of the SNPs tree groups with the virulence factors groups

**Fig. 10:**
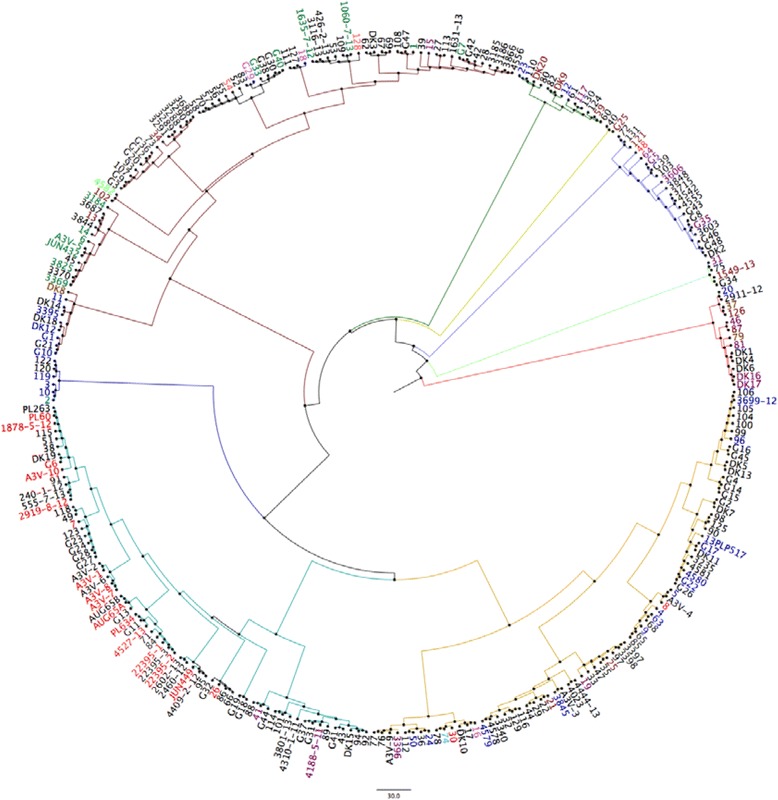
Best reciprocal Blast hits analysis: cluster tree. Each sample name is composed by: name, SNPs group, Country of origin, Rtype(s), phylogroup (e.g. A3V5 7 IT R4 D = A3V5(name) 7(SNPs) IT (Country), R4 (Rtype) D (phylogroup))

A comparison of the group data obtained with the whole genome SNPs analysis with the multiplex PCR generated virulence tree was done. This analysis identified an overall convergence of the strains (selected from each SNPs group) into the groups obtained with the multiplex PCR as reported in Fig. 10. From this tree it is apparent that, with a few exceptions, the SNPs group 1 (group to which O78/ST23 belong) (red text) is mainly located in the PCR group 7 (turquoise branch) in its second subgroup. In the same way SNPs group 7 (O4, O24, O35, O53, O161/ST117) (dark green text) is mainly located in the group 9 (subgroup 2) (dark red branch). However, SNPs group 8 (different serotypes/different STs) (dark blue text) was split between groups 1, 3, 5, 6, 8 and 9.

A possible explanation for why this group (and other SNPs groups) did not merge into a one single group could be due to the variability (plasticity) of the genome of these APEC strains and specifically with regards to the genes analysed using the multiplex PCR assay (used to generate the virulence tree) [58]. However, when the whole genome is taken into consideration, these differences can be seen as minor changes in the overall genome similarity (so they group together in the SNPs tree).

It is also interesting to note that the multiplex groups 1 (dark green), 2 (yellow), 3 (dark blue), 4 (light green) and 5 (red) are not only very distant from the other groups, but they are also hyper-variable strains that belong to different groups when analysed using SNPs. This opens up a different hypothesis: these strains could be ancestors of other, more frequently isolated strains, or they could be newly emerged strains currently expanding clonally or they can be just transient types.

#### Genome similarity analysis of strains

**Fig. 11:**
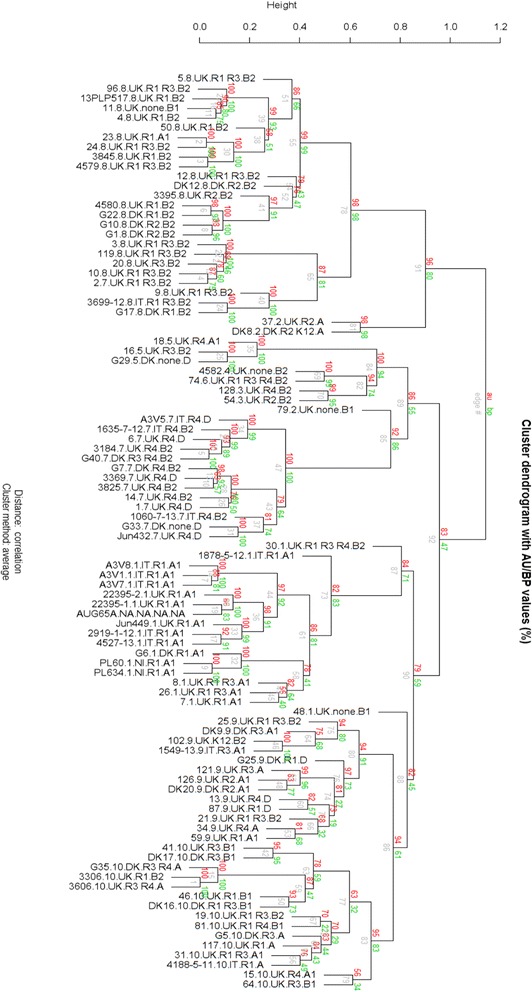
Strains selection criteria. Strains were selected according to their virulence tree group, country of provenience, R-type, phylogroup, host species, broiler/layer (B,L, ? (unknown), N.A. Not Applicable) and clinical symptoms reported

Best reciprocal Blast hits approach confirmed the correctness of the SNPs tree giving comparable results. As it can be seen in Fig. 11, the convergence of SNPs groups into the groups generated by best reciprocal Blast hits analysis is, with only a few exceptions, complete. The groups matching can be observed also in the heat map (Additional files 3 and 4) where SNPs groups have been highlighted (black boxes).

The heat map generated from the similarity matrix (Fig. 6) allowed a similarity comparison between the 10 groups identified with SNPs analysis. Using this tool it can be noticed all strains are divided in three similarity groups that we called macro-groups 1, 2 and 3. While macro-groups 1 and 3 are composed by only few SNPs groups (see Fig. 10), macro-group 2 contains, together with the other SNPs groups that were not present in the other macro-groups, isolates from SNPs groups that actually were represented in macro-groups 1 and 3. This finding could suggest that the strains in macro-group 2 could be either ancestors or newly evolved (recombinant?) strains. The greater number of isolates belonging to macro-group 1 and 3 may suggest that those strains (and SNPs groups) are the ones that developed a more efficient host/pathogen/environment relationship and are, evolutionary speaking, successful.

## Conclusion

The 10 groups identified using whole genome SNPs analysis were confirmed using different approaches and in all cases the data obtained with these analyses (PCR, MLST, Best reciprocal Blast hits) were comparable with the groups obtained with the SNPs analysis. This confirmed that the whole procedure adopted (choice of the samples using PCRs results, *de novo* assembly, SNPs analysis) was justified.

However, previous authors [41, 42, 45] have found that different phylogroups can harbour varying numbers of virulence genes, in the study presented here this was not always the case (Table 8). Perhaps this could be due to the particular nature of the samples analysed (they were all confirmed as disease causing strains in poultry).

**Table 8:** Contingency table between Phylogroup and number of virulence factors. Chi-square test; *p*-value = 0.0325

	Number of virulence factors										
		1	4	5	6	7	8	9	10	11	12
	A	1	0	0	1	3	1	2	1	0	0
Phylogroup	A1	0	1	1	4	2	6	8	3	0	0
	B1	0	2	2	1	1	2	0	0	0	1
	B2	0	2	0	6	8	5	15	3	3	0
	D	0	0	1	0	4	2	2	0	1	0

Interestingly, whilst in Germany the phylogroups found were almost equally distributed (with B2 the most prevalent 35.3%) in Italy there is a prevalence of the phylogroups A1 (53.3%) and B2 (33.3%) and in the UK there is an overwhelming presence of the group B2 (56.1%) among the other phylogroups.

However, further work has to be done to confirm statistically the geographical distribution of serotypes and MLST. The results so far may indicate a higher variability of serotypes and MLST in the UK compared with and Italy. If this is confirmed by further analysis of evenly distributed samples, it could reflect the different numbers of farms (different companies) involved in great-grandparent production in those Countries. In fact, according to the data reported in the European final report “Study of the impact of genetic selection on the welfare of chicken bred and kept for meat production” (SANCO/2011/12254), most sites and birds are located in the UK and France (65–90 sites 1,200 K-1,400 K birds) followed by Germany and the Netherlands (35–50 sites 900 K-1,100 K birds), Ireland, Spain (15–20 sites 300–450 k birds) Hungary, Sweden (10–15 sites 150–300 K birds), Denmark, Finland, Poland (5–10 sites 50–200 K birds) Belgium, Czech Republic, Italy (<5 sites <50 K birds). Knowing these data, it could be reasonable to make the hypothesis that a greater variability in serotyping and MLST could be due to the different great-grand parent structures that are present in the UK, Germany and Italy. APEC could be transmitted from their great-grand parents, following the whole chicken production line, to the broilers. Unfortunately, for commercial reasons, the data regarding the breeders have been omitted from this report, hence it is not possible to correlate specific serotypes or MLSTs to a particular breeder or production management type. This hypothesis is in line with the AMR findings of Obeng et al. (2011) that noticed the lack of significant difference between intensive and free-range chickens because free-range chicken producers in their study were supplied from the same hatcheries as intensive producers and they conclude that this may indicate that resistance genes (hence any gene) could be passed vertically from breeder flocks [59, 60].

The higher variety of serogroups reported here (*n* = 23) may be due to the *in silico* analysis that was able to detect the different serogroups unbiased from cross-reactions that can happen in a laboratory environment. The downside of the *in silico* method is that eventual punctual mutations (or minor mis-assembly) may give as result an ‘un-typable” isolate.

In total 34 different ST types were found and 10 strains were un-typable (data available in supplement (Additional file 2)). This result expanded the finding of Olsen et al. where they found just eight different ST types [61]. Also in this case it could be possible to apply the considerations done for the *in silico* serotyping.

When analysed by country the AMR data (in the Additional file 2), it was evident that the German strains were more frequently sensitive to antibiotics (72.2%) compared to UK (49.2%) and Italy (25%) possibly indicating different management systems for antibiotic use in German farms.

Using the diagnostic tests developed in this study (multiplex PCRs) it will be possible to provide clear guidance about the potential pathogenic potential of the APEC analysed. This type of data will facilitate the provision of tailored advice regarding preventive/therapeutic measures that should be adopted.

The very nature of data mining and machine learning concept is that rules (and associations) are found empirically from a dataset, so larger datasets could result in different results. For this reason, these associations may indicate co-selection and it would be of interest to determine the physical relationship between the genes in these gene pairs/sets. One hypothesis is that these may be co-located either on the same whole genome backbone or perhaps co-located on transient plasmidic DNA, but further analyses is required to confirm this. We suggest the data generated here are biologically meaningful and encourage the analysis approach be employed for the interrogation of large datasets, such as those presented in this study.

## Additional files

Additional file 1: Assembly statistics. (XLSX 51 kb)
Additional file 2: Cumulative table. (XLSX 87 kb)
Additional file 3: Hits. (XLSX 2118 kb)
Additional file 4: Heatmap_hit_grouped. (PDF 1530 kb)

## Abbreviations

AHT: Animal health trust
AMR: Antimicrobial resistance
APEC: Avian pathogenic Escherichia coli
BRIG: Blast ring image generator
LPS: Lipopolysaccharides
MDR: Multi drug resistance
MLST: Multilocus sequence typing
NGS: Next generation sequencing
PCR: Polymerase chain reaction
QRDR: Quinolone resistance-determining regions
SNPs: Single-nucleotide polymorphisms
WGS: Whole genome sequencing
