# Supplementary figures and images for: Comparative genomics of European avian pathogenic E. Coli (APEC)
Source: BMC Genomics. 2016 Nov 22;17:960. doi: 10.1186/s12864-016-3289-7 (PMC5120500; doi:10.1186/s12864-016-3289-7)

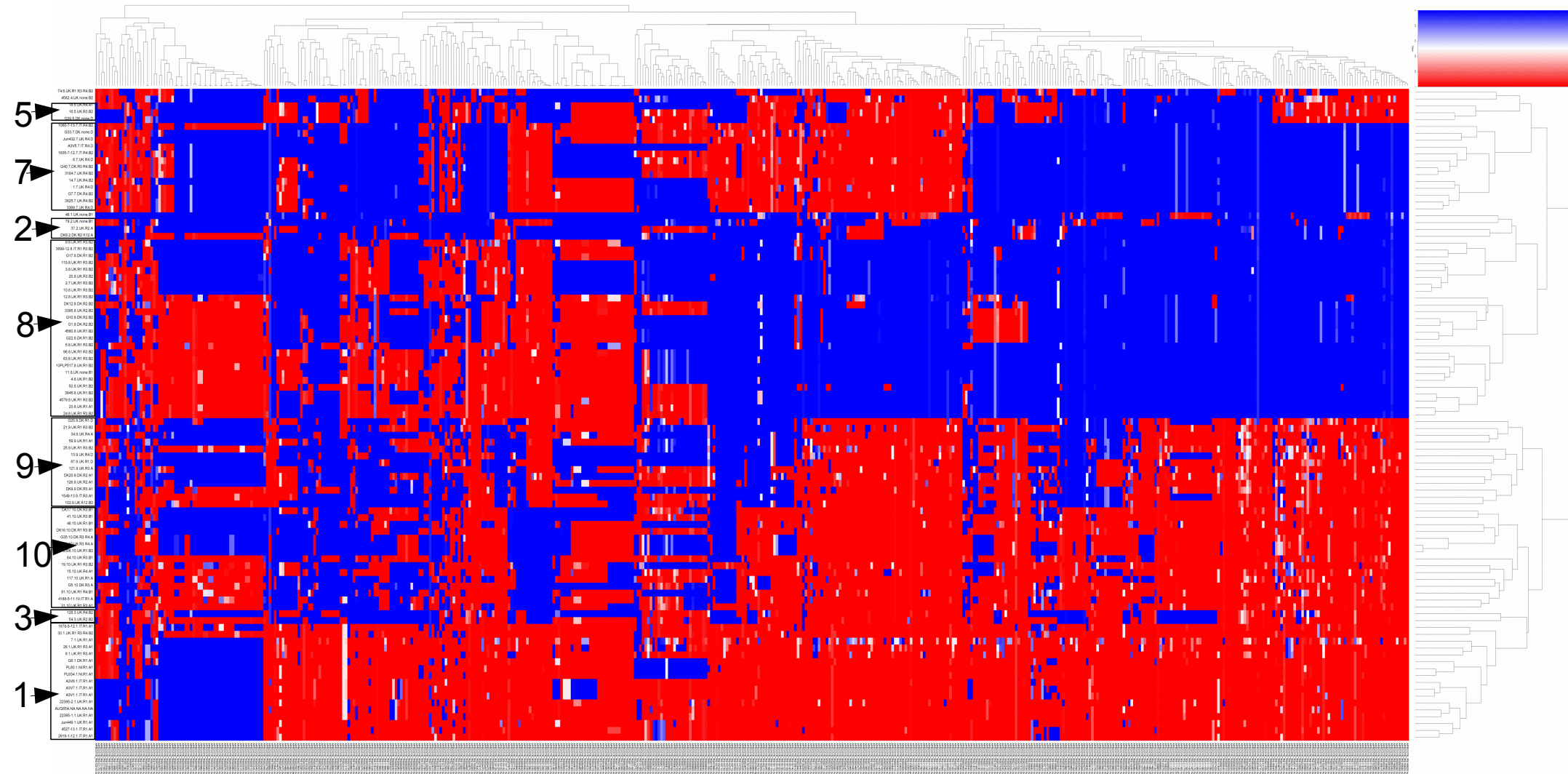

Supplement: Additional file 4: — Heatmap_hit_grouped. (PDF 1530 kb) [file 12864_2016_3289_MOESM4_ESM.pdf]
